# Self-Sensing Properties of Alkali Activated Blast Furnace Slag (BFS) Composites Reinforced with Carbon Fibers

**DOI:** 10.3390/ma6104776

**Published:** 2013-10-22

**Authors:** Josep Lluís Vilaplana, Francisco Javier Baeza, Oscar Galao, Emilio Zornoza, Pedro Garcés

**Affiliations:** Civil Engineering Deparment, University of Alicante, Ctra. San Vicente s/n, San Vicente del Raspeig 03690, Spain; E-Mails: joseplluisvilaplana@citop.es (J.L.V.); fj.baeza@ua.es (F.J.B.); oscar.galao@ua.es (O.G.); emilio.zornoza@ua.es (E.Z.)

**Keywords:** strain sensing, damage sensing, alkaline cement, carbon fibers, multifunctional composites

## Abstract

In recent years, several researchers have shown the good performance of alkali activated slag cement and concretes. Besides their good mechanical properties and durability, this type of cement is a good alternative to Portland cements if sustainability is considered. Moreover, multifunctional cement composites have been developed in the last decades for their functional applications (self-sensing, EMI shielding, self-heating, *etc.*). In this study, the strain and damage sensing possible application of carbon fiber reinforced alkali activated slag pastes has been evaluated. Cement pastes with 0, 0.29 and 0.58 vol % carbon fiber addition were prepared. Both carbon fiber dosages showed sensing properties. For strain sensing, function gage factors of up to 661 were calculated for compressive cycles. Furthermore, all composites with carbon fibers suffered a sudden increase in their resistivity when internal damages began, prior to any external signal of damage. Hence, this material may be suitable as strain or damage sensor.

## 1. Introduction

Portland cement production consumes large amounts of energy, and is responsible for large emissions of sulphur and nitrogen oxides, besides CO_2_, to the atmosphere. Nowadays, inorganic industrial products, which show pozzolanic properties, have been widely investigated as an alternative to Portland cement for cement composites [1,2,3,4,5,6,7,8,9].

Therefore, environmental and economic reasons make the alkaline activation of granulated blast furnace slag (BFS) appear to be a great alternative to the conventional production of Portland cement. Actually, the complete replacement of Portland cement by alkaline cement could be possible.

The use of waterglass (sodium silicate base activators, Na_2_SiO_3_•*x*H_2_O + NaOH) as an alkaline activator for BFS has been recommended for the mechanical and durability properties of their composites [1], especially for their resistance against aggressive agents like sulphates and chlorides. Low porosity, high compressive strength, low hydration heat, high carbonation resistance or high freeze-thaw resistance are some of the properties shown by these composites [2,3,4,5,6,7,8,9].

On other hand, the development of multifunctional materials has been investigated over the last decades to fulfill the demand for smart structures, capable of being sensitive and responding properly to certain stimuli. These functional properties (e.g., strain or damage sensing, temperature sensing, heating control, damping, electromagnetic waves reflection and absorption or anode for electrochemical chloride extraction) are the future of new construction materials, where both mechanical properties and functional applications are shown by only one material [10,11,12,13,14,15,16,17,18].

To achieve these functionalities, a certain level of electrical conductivity is necessary. Therefore, as concrete is a bad electrical conductor, conductive admixtures are needed. Several researchers have focused on these conductive admixtures (e.g., steel fibers, or carbon materials: carbon fibers, graphite powder, carbon nanofibers or nanotubes) in order to achieve a better electrical behavior without compromising the composite’s mechanical properties [12,13,14,15,16,17,19,20,21,22,23,24]. Thus, for each type of conductive admixture, the relationship between their dosage and the composite’s conductivity has to be determined. Researchers have been interested in the minimum amount of admixture which guarantees a low material’s resistivity. In this regard, percolation could be defined as the situation where the conductive fibers or particles, randomly dispersed, touch and continuous electrical paths along the material appear [25]. The minimum admixture dosage that creates these conductive pathways is known as the percolation threshold, as reported by several authors working with different admixtures and matrices [25,26,27,28].

When the strain sensing function is characterized in a cementitious material, the response on the volumetric electrical resistivity (proportional and reversible) related to its strain state has to be defined. For example, if a longitudinal compressive stress is applied to the material, then the electrical resistance on that direction is reduced. However, if the specimens were upon tension the effect would be the contrary. Both effects are reversible in the elastic range of the material,* i.e.*, the composite’s electrical resistance recovers its initial value once the load is removed. The damage sensing mechanism explores the plastic range of the material,* i.e.*, once the yielding point is exceeded. This main behavior characteristic is related to the irreversible changes on the electrical resistivity, which can be observed as the material’s stresses and is close to its ultimate strength.

Strain and damage sensing have been investigated in several research papers focused on fiber-reinforced cementitious materials based on Portland cement. Nonetheless, these applications of carbon fiber-reinforced cement composites based on alkaline activation have not yet been studied. However, there are good results regarding the use of carbon fibers as addition in alkaline cements, especially improving their shrinkage behavior [6].

The main objective of the present paper is to evaluate the strain and damage sensing properties of carbon fiber-reinforced alkaline cement pastes. Thus, the multifunctionality of carbon fiber reinforced cement composites (CFRCC) would be improved by the sustainability of alkaline cements.

## 2. Experimental Program and Materials.

### 2.1. Materials and Sample Fabrication

Prismatic specimens of dimensions 40 × 40 × 160 mm^3^ were fabricated. Carbon fiber alkaline cement pastes were made using blast furnace slag (BFS) with an alkaline activator compound of commercial waterglass (27% SiO_2_, 8% Na_2_O and distilled water) and a NaOH solution. The alkali modulus of the activator (SiO_2_/Na_2_O ratio) was 1.2, and a 5% Na_2_O concentration (by BFS mass) was used in order to obtain an activator/BFS ratio of 0.43. The chemical composition of BFS as provided by the supplier (ENSIDESA factory-Avilés Spain) is: 41.00% CaO, 35.54% SiO_2_, 13.65% Al_2_O_3_, 4.11% MgO, 0.39% Fe_2_O_3_, 0.06% SO_3_^−^, 1.91% S_2_^−^, 0.01% Na_2_O_2_, 0.00% free CaO, 2.72% lost on ignition and 0.64% insoluble residue; furthermore the BFS vitreous phase was 99% and its specific surface was 325 m²/kg. Carbon fibers (CF) type PANEX 35 (supplied by Zoltek Rt) were added as conductive admixture (their main properties are included in Table 1). Three different CF dosages were prepared: 0%, 0.38% and 0.76% by BFS mass, which correspond to volumetric fractions of 0, 0.29 and 0.58 vol %.

**Table 1:** Carbon fiber PANEX 35 properties.

Property	Value	Unit
Diameter	7.2	µm
Length	3.5	mm
Carbon content	95	%
Tensile strength	3800	MPa
Elastic modulus	242	GPa
Resistivity	1.52 × 10^−3^	Ω·cm
Density	1.81	g/cm^3^

Two different treatments were applied to CF to improve their dispersion in the mix. First of all, an oxidation treatment was conducted by placing the fibers in air at 400 °C with an air flow of 10 mL/min for 4 h [29]. Afterwards, the oxidized CF was stirred by hand in the activator and an ultrasounds treatment was applied for 10 min [13]. Then all materials were mix for five minutes and the fresh mix was then poured into prismatic steel molds. After applying a mechanical treatment to remove any entrapped air, the molds were kept in controlled environment room (20 °C and >99% RH) for 24 h. Afterwards, specimens were demolded and conserved in the same conditions until a curing age of 28 days. After the first 28 days, ambient conditions (ambient temperature and 100% RH) were maintained for a total of 90 days curing period in saturated conditions.

Prior studies on strain sensing function in Portland cement pastes concluded that the highest sensitivities were obtained for samples in non-saturated conditions [10,13,19]. Therefore prior to the self-sensing tests (damage or strain), all samples were partially dried at 50 °C for 26 days. After this process, the specimens’ average mass loss was 5% of their initial mass. Finally, samples were tested at an age of 120 days.

**Figure 1:**
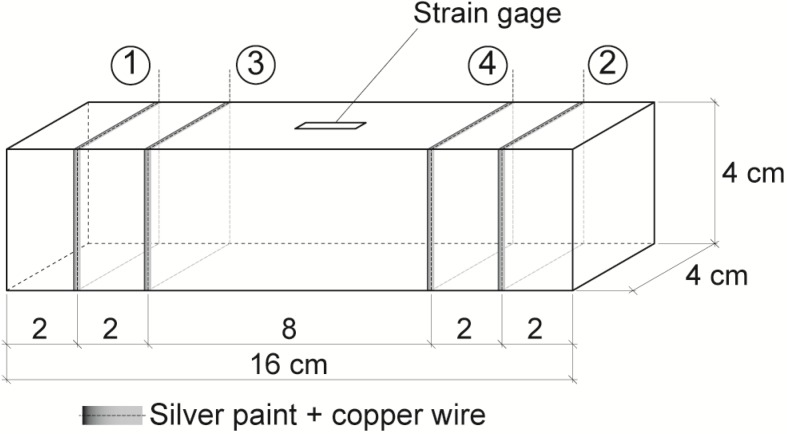
Electrical contacts: 1 and 2 are for current input; 3 and 4 for voltage measurement.

Before testing, the perimeter at four interior planes, which were parallel to the end surfaces, was painted with electrically conductive silver paint (Pelco conductive Silver 187). Around each silver painted perimeter a copper wire was wrapped, in order to form the electrical contacts, as needed for the four-probe method of electrical resistance measurement. The position of each electrical contact is included in Figure 1.

Electrical current intensity input to take electrical resistance measures during sensing test (strain or damage), was fixed with an AC/DC current source (model Keithley 6220) and passed between the outer contacts (1 and 2 in Figure 1), while the voltage was measured between the inner contacts (3 and 4 in Figure 1) using a digital multimeter (model Keithley 2002). Electrical resistance may be calculated applying Ohm’s law. All tests were made on an electromechanical press with loading cells with a maximum load of 20 kN for strain sensing tests and 100 kN for damage sensing (until failure). Longitudinal strain was permanently registered with a Vishay P3 extensometer and strain gages were attached to the middle point of one lateral side of each specimen.

### 2.2. Strain Sensing Tests

Strain sensing tests, consisted on consecutive compressive loading-unloading cycles applied in the specimen’s longitudinal direction. The effect of different variables was studied, e.g., the maximum cycle’s load and the percolation level,* i.e.*, the sample’s CF dosage. The aim of this test is to relate the fractional change in longitudinal resistance to the longitudinal strain. Strain sensing sensitivity is typically measured using the gage factor (GF), which is defined as the fractional change on the electrical resistance per strain unit. This parameter can be calculated according to Equation (1) [20,26].

(1) GF=∆ρρ0∆ll0=∆RR0ε

where: ∆*R*: change on electrical resistance; R_0_: initial electrical resistance; ∆*l*: longitudinal deformation; *l*_0_: initial length; *ε*: longitudinal strain.

The maximum compressive stress of each cycle was 1.25, 2.50, 3.75, 5.00 and 7.50 MPa, which correspond to load values of 2, 4, 6, 8 and 12 kN respectively. Loading rate was fixed at 200 N/s according to previous researches [13,19].

### 2.3. Damage Sensing Tests

Once the strain sensing work plan was performed, all specimens were loaded up to its ultimate strength. The testing procedure was similar to the strain sensing one. However, in this case the maximum load was progressively increased after each cycle, until failure was reached. The maximum compressive stress of each cycle was 2.5, 5.0, 7.5, 10.0, 12.5, 25.0 MPa and then it was increased by 25 MPa per cycle. The loading rate in these tests was also 200 N/s up to 25 MPa and 400 N/s afterwards.

The aim of these tests was to note if the specimens were capable of sensing their own structural damage. If a damage sensing mechanism was triggered then a safety time-range before a collapse could be determined.

## 3. Results and Discussion

### 3.1. Compressive Strength and Electrical Characterization

Initially, compressive strength tests were made to determine the loading limits for the strain sensing tests, with 2 control specimens (0% CF by BFS mass). In order to guarantee an elastic behavior, the loading conditions during stain sensing tests should be below 30% of compressive strength (according to Spanish Standard UNE 83316:1995).

Compressive strength values of alkaline activated BFS were measured according to UNE-EN 1015-11:2000/A1:2007. Two samples of the control dosage (without CF) conserved in 100% RH ambient were tested at a curing age of 120 days. Their compressive strengths were 107.44 and 106.61 MPa.

The conductivity of cement composites can be easily improved with the addition of conductive admixtures, as mentioned above. Table 2 includes the electrical resistance and resistivity results for all three CF dosages. As expected, a higher amount of CF in the mix resulted in a higher increase of the composite’s conductivity,* i.e.*, resistivity values of 10.1 × 10^3^, 97.86 and 9.95 Ω·cm for a control sample (with no CF), a partially percolated paste (0.29 CF vol %) and a percolated paste (0.58 CF vol %), respectively. If these results are compared to the resistivity measured in Portland cement pastes, similar trends are shown. Portland cement pastes with CF volumetric fraction of 0.28 vol % and 0.56 vol % showed resistivity values of 462.55 and 31.02 Ω·cm respectively [21]. Therefore, for the same fiber’s dosage, the alkaline cement composites showed higher levels of conductivity. Similar conclusions regarding the percolation phenomena could be applied.

**Table 2:** Electrical resistance and resistivity values for different carbon fibers (CF) dosage by BFS mass.

CF mass %	CF vol %	Resistance (Ohm)	Resistivity (Ohm·cm)
0%	0%	5.06 × 10^3^	10.1·× 10^3^
0.38%	0.29%	48.93	97.86
0.76%	0.58%	4.98	9.95

### 3.2. Strain Sensing Test

**Figure 2:**
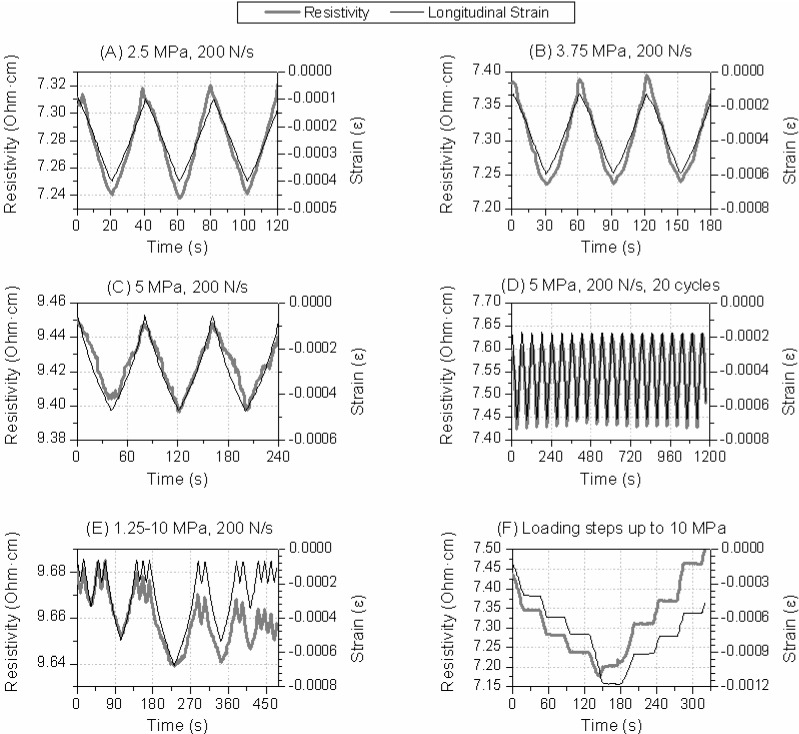
Resistivity and longitudinal strain data* versus* time for different samples of a BFS composite with 0.76% CF (by BFS mass). Loading rate was 200 N/s in all tests, and the maximum compressive stress was change between tests.

In order to characterize the strain-sensing function, the influence of different variables was studied. Typical parameters such as maximum load level or loading rate were first tested [20]. Moreover, other aspects like the way of applying the compressive stress were also evaluated, e.g., loading and unloading cycles between the same stress levels, alternating cycles with different maximum load values, or even if the material’s response was stable when a constant load was maintained for several seconds, were compared. All different types of tests are summarized in Figure 2 where the measured strain and resistivity are both plotted* versus* time for 0.76% CF by BFS mass specimens.

Figure 2A–C show the response of a composite with 0.76% CF (by BFS mass) for three different stress levels. For all specimens and loading levels a correlation between electrical resistivity and longitudinal strain can be observed,* i.e.*, when compressive longitudinal strains increased, a decreased in the resistivity was measured, which was reversed as the load was removed. Thus, the strain sensing function was detected for BFS composites. Figure 2D includes the results (resistivity and strain values) for a 20-cycle test in order to prove the phenomenon’s repeatability. In this case, the composite’s response did not change between cycles for a 0.58 CF vol % composite. In the last type of tests, included in Figure 2E,F, stresses were changed between cycles, and were increased up to 7.5 MPa. In these cases an irreversible response in the specimens’ resistivity was detected. This irreversibility can be easily observed in Figure 2F, where the same loading level showed different resistivity values before and after the maximum stress had been applied. Similar conclusions could be drawn for the results shown in Figure 2E.

**Figure 3:**
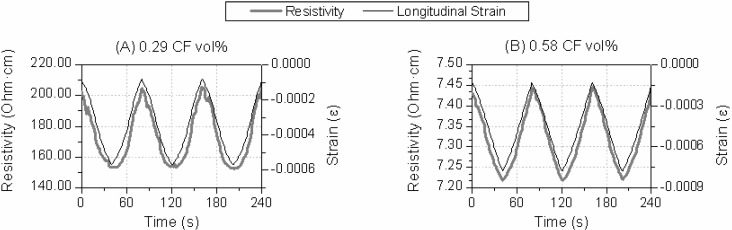

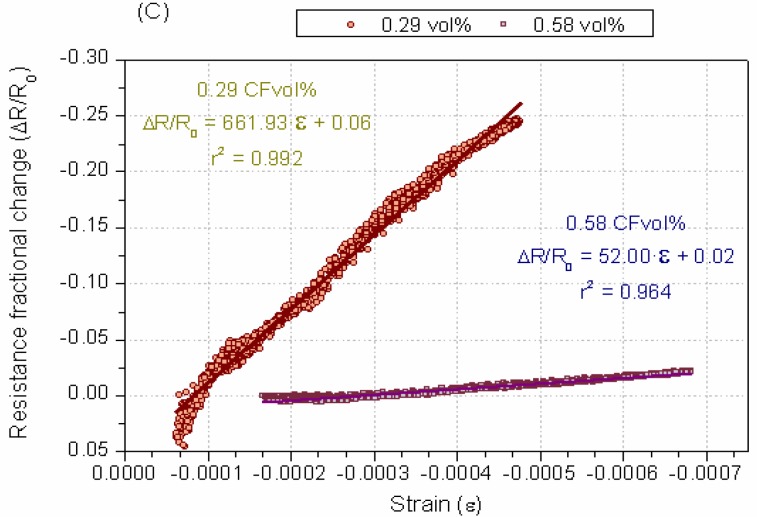
Effect of carbon fiber addition: resistivity and strain* versus* time for a 5 MPa test and (**A**) 0.29 CF vol %; (**B**) 0.58 CF vol %; (**C**) Fractional change of resistance* vs.* longitudinal strain for 20 loading-unloading cycles tests, also linear regression functions are represented.

The next variable of study was the CF dosage. Figure 3 includes test results for different CF additions at the same test conditions (8 kN, 5 MPa and 200 N/s). Both samples showed self-sensing behavior,* i.e.*, relationship between resistivity and strain. In order to evaluate each composite’s sensitivity, the curve included in Figure 3C was drawn for the 20-cycle test and gage factors (GF) were calculated according to Equation (1). The resistivity fractional change* versus* longitudinal strain is included in Figure 3C for both dosages. A linear relationship between both variables was registered, as shown by the linear regression functions (*r*^2^ Pearson’s coefficients higher than 0.96). The materials’ response was stable during all 20 cycles. The higher CF addition did not guarantee a better sensitivity. Actually the calculated GF were 661.9 and 52.0 for the 0.29 and 0.58 CF vol %, respectively. These results are better than the ones obtained for Portland cement pastes, where GF of around 40 were calculated for 0.28 CF vol % [21].

### 3.3. Damage Sensing Results

Previous research using Portland cement pastes reported a non-linear electrical response when specimens’ damage begun,* i.e.*, if permanent strains had been induced, irreversible changes in resistivity would have occurred. This permanent effect on composite’s resistance was more noticeable as the maximum stress was increased [13,19]. Hence, this damage sensing tests explored the material behavior in the plastic range.

**Figure 4:**
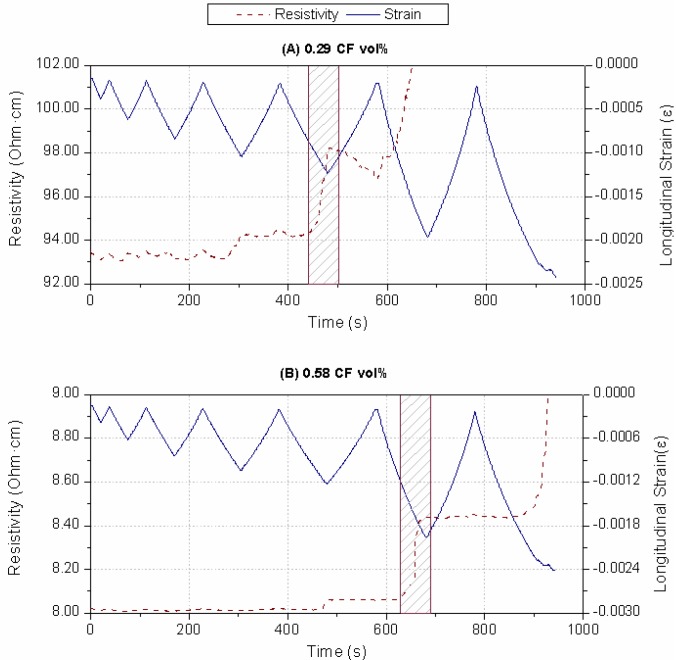
Fractional change of resistance and longitudinal strain* versus* time until materials failure for different CF additions: (**A**) 0.29 CF vol %; (**B**) 0.58 CF vol %. The shaded area corresponds to the damage sensing mechanism activation.

Figure 4 shows the resistivity and strain data registered during damage sensing tests for both CF amounts. In all cases, a sudden increase in the specimen resistivity was detected prior to failure,* i.e.*, electrical resistance increases abruptly when damage is produced. Afterwards, the expected electrical behavior (as explained for strain sensing) is affected, and during the following unloading cycle the electrical resistance decreases instead of increasing, as shown in Figure 4A for a 0.29 CF vol % specimen.

In prior studies, this damage-sensing function was studied for CF composites in a Portland cement matrix [19]. In these CF cement pastes, a good strain sensing capacity was shown, even if high damage levels were experienced. Besides, there was not an abrupt increase in the composite’s resistivity, in contrast to the curves included in Figure 4. However, the most important aspect of alkaline cement pastes’ behavior is that the material warning (*i.e*., the high increase in its resistivity) occurred before any external evidence of failure could be seen, and the material was capable of resisting higher stresses.

## 4. Conclusions

Alkali activated blast furnace slag composites were fabricated with different carbon fiber dosages. After casting strain and damage sensing tests, the following conclusions can be drawn.

These materials showed strain and damage sensing properties. Therefore, they could be suitable as strain sensors or even sensitive to their own structural damage before any external evidence of failure had been observed.

Higher amounts of CF decreased the composite’s electrical resistivity in several orders of magnitude. However, the highest gage factor values during strain sensing tests were calculated for lower CF additions,* i.e.*, for composites with lower conductivity. These gage factors, obtained for an alkali activated BFS composite, were higher than those measured in similar Portland cement pastes.
